# Degenerate minigene library analysis enables identification of altered branch point utilization by mutant splicing factor 3B1 (SF3B1)

**DOI:** 10.1093/nar/gky1161

**Published:** 2018-11-20

**Authors:** Abhishek K Gupta, Tushar Murthy, Kiran V Paul, Oscar Ramirez, Joseph B Fisher, Sridhar Rao, Alexander B Rosenberg, Georg Seelig, Alex C Minella, Manoj M Pillai

**Affiliations:** 1 Section of Hematology, Yale Cancer Center, New Haven, CT, USA; 2 Driskill Graduate Program, Northwestern University Feinberg School of Medicine, Chicago, IL, USA; 3 Blood Research Institute, BloodCenter of Wisconsin, Milwaukee, WI, USA; 4 Department of Electrical Engineering, University of Washington, Seattle, WA; 5 Paul G. Allen School of Computer Science and Engineering, University of Washington, Seattle, WA, USA

## Abstract

Cancer-associated mutations of the core splicing factor 3 B1 (SF3B1) result in selection of novel 3′ splice sites (3′SS), but precise molecular mechanisms of oncogenesis remain unclear. SF3B1 stabilizes the interaction between U2 snRNP and branch point (BP) on the pre-mRNA. It has hence been speculated that a change in BP selection is the basis for novel 3′SS selection. Direct quantitative determination of BP utilization is however technically challenging. To define BP utilization by SF3B1-mutant spliceosomes, we used an overexpression approach in human cells as well as a complementary strategy using isogenic murine embryonic stem cells with monoallelic K700E mutations constructed via CRISPR/Cas9-based genome editing and a dual vector homology-directed repair methodology. A synthetic minigene library with degenerate regions in 3′ intronic regions (3.4 million individual minigenes) was used to compare BP usage of SF3B1^K700E^ and SF3B1^WT^. Using this model, we show that SF3B1^K700E^ spliceosomes utilize non-canonical sequence variants (at position −1 relative to BP adenosine) more frequently than wild-type spliceosomes. These predictions were confirmed using minigene splicing assays. Our results suggest a model of BP utilization by mutant SF3B1 wherein it is able to utilize non-consensus alternative BP sequences by stabilizing weaker U2-BP interactions.

## INTRODUCTION

Mutations in the HEAT domains of the core splicing factor 3 B1 (SF3B1) are common in several malignancies including myelodysplastic syndrome (MDS) (1,2), chronic lymphocytic leukemia (CLL) (3), uveal melanoma (4) and cancers of breast (5), liver (6) and pancreas (7). These mutations are non-synonymous and mutually exclusive with other splicing factor mutations (in U2AF1, SRSF2 and ZRSR2) suggesting their role as driver mutations with neomorphic functions (8). Transcriptomic profiling of clinical samples, cell lines and isogenic murine models have shown that the primary splicing defect in SF3B1-mutant cells is the use of novel or ‘cryptic’ 3′ splice sites (3′SS) (9–13). The precise mechanisms by which mutant spliceosomes alone recognize these cryptic 3′SS is unclear. Given the purported function of SF3B1 in stabilizing the interaction between U2 snRNP and branch point (BP) on the pre-mRNA (14), it has been speculated that these mutations alter BP utilization and consequently the 3′SS (10, 15). Genome-wide and targeted mapping of BP in isogenic SF3B1-mutant cell lines however failed to reveal novel BP exclusive to SF3B1-mutant cells (16). Alternate models of novel 3′SS selection without obligate change in BP have been proposed wherein mutant spliceosomes overcome steric protection of 3′SS or recognize 3′SS within secondary structures (9,11).

BP utilization in SF3B1-mutant cells is difficult to precisely determine for several reasons. Computational prediction of vertebrate BP is difficult given lack of clear consensus sequence (17). BP-containing intronic intermediaries (called lariats) are not present in detectable frequencies in routine RNA-Seq libraries to directly determine BP (16,18,19). Genome-wide analysis of BP (through isolation and sequencing of lariats) is feasible, but technically challenging (18). Finally, total numbers of cryptic 3′SS (and associated BP) in patient-derived datasets are typically no >1000 (9–11,15,20) (compared to ∼350 000 3′SS from the annotated splice junctions in the human hg19 genome build), which limits their utility in statistical analysis and computational modeling.

To better define BP in SF3B1-mutant cells we utilized two experimental systems: one employing overexpression of mutant versus wild-type SF3B1 in a transformed, human cell line (HEK293T) and hemizygous *Sf3b1*-mutant, isogenic, murine embryonic stem cells (mESC), generated using CRISPR/Cas9 gene editing. Isogenic cells offer the advantage of a physiological gene-dose given the hemizygous nature of disease-associated SF3B1 mutations. We then used a massively parallel reporter assay (MPRA) in which a synthetic minigene library with degenerate sequences upstream and downstream of an alternate 3′SS was transfected in wild-type (SF3B1^WT^) and mutant SF3B1 (mutated at position 700 from lysine to glutamic acid, or SF3B1^K700E^) cells. Comparative analysis of the minigene transcriptomes revealed key differences in how SF3B1^WT^ and SF3B1^K700E^ utilize BP sequences. Our results show that while both SF3B1^K700E^ and SF3B1^WT^ prefer canonical BP, SF3B1^K700E^ utilizes non-canonical BP sequences (with non-consensus nucleotides at −1 positions relative to BP adenosine) more often than SF3B1^WT^. These data also support a biochemical mechanism for altered 3′SS wherein SF3B1^K700E^ accommodates weaker U2-BP interactions during spliceosome assembly.

## MATERIALS AND METHODS

### Molecular Cloning, cell culture, transient transfection, genome editing and next generation sequencing

Codon-optimized Flag-tagged wild-type(WT) and K700E open reading frames of SF3B1 were cloned into pCDNA3.1+ (Addgene #82576, #82577 (11)). HEK293T cells were grown in DMEM media supplemented with 10% (v/v) fetal bovine serum (FBS) and 1% (v/v) penicillin/streptomycin. Cells were transiently transfected with plasmid using Lipofectamine 2000 reagent (Life Technologies). Briefly, 50 000 cells were seeded per well of 24-well plate 24 h prior transfection in antibiotic free media and transfected with plasmid using 7.5 ul of Lipofectamine 2000. Optimal concentration of SF3B1 plasmid was determined by western blot (Supplementary Figure S1A and B). For co-transfections of FLAG-SF3B1 and 3′SS library, 2.5 ug total plasmid (250 ng SF3B1 plasmid + 2250 ng of 3′SS library) were used. Cells were harvested 48 h after transfection to isolate protein or total RNA.

CCE mouse embryonic stem cells (mESC) were a kind gift of Dr Mitchell Weiss, St. Jude's Children's Hospital, Memphis, TN, USA) and were cultured on tissue-culture dishes coated with 0.1% (w/v) gelatin in DMEM media supplemented with 15% (v/v) fetal bovine serum, 1% (v/v) penicillin/streptomycin, 1% (v/v) GlutaMAX, 0.4 mM 1-thioglycerol and leukemia inhibitory factor (LIF). Cells were transfected with indicated plasmid vectors using Lipofectamine 2000 Reagent. Drug selection was performed with puromycin (2 μg/ml) for 48 h and Hygromycin-B (250 μg/ml) or Neomycin (350 μg/ml), both for 10 days. mESC were transfected with 3′SS plasmid library as described for HEK293T cells.

Guide RNA (sgRNA) (5′GCTCAAGCCCCTATGGA3′) for targeting mouse *Sf3b1* locus was designed using the Zhang Lab CRISPR design tool (crispr.mit.edu). sgRNA was cloned into the BbsI restriction site of pX459-V2 ((21), Addgene #62988). Repair vectors were generated by cloning homology arms into the SalI–EcoRI and BamHI–NotI restriction sites of pL452-Neo (22) and pL452-Hygro vector was generated by replacing the Neomycin resistance gene within pL452-Neo with a Hygromycin resistance gene using BclI and BsmI restriction sites. See Supplementary Methods for more details.

### 3′SS minigene library, next generation sequencing and bioinformatics analysis

3′SS library has been previously described (23). Preparation of the next generation library and bioinformatics analysis are detailed in Supplementary Methods.

### Minigene assay and RT-qPCR

Minigene constructs used were based on the original 3′SS plasmid vector described previously (23). The region including two exons of citrine, the single intervening intron and 3′ UTR were PCR amplified and cloned into pCDNA 3.1 (cloning strategy detailed in Supplementary methods). The base minigene vector had a BP sequence CTAAC placed 29 bp upstream of SA1 and was noted to utilize both SA1 and SA2 (Figure 4A and B). Minigene variants (variations at −1, −2 and 0 positions with respect to the nucleophilic adenosine) were constructed from this base vector. Individual minigenes were co-transfected with SF3B1 plasmid (wild-type or K700E) followed by extraction of RNA and reverse transcription as described for 3′SS libraries above. For negative control (–RT) no reverse transcriptase was added. Resulting cDNA was amplified using forward (F1) and reverse (R1) to detect the splicing isoforms SA1 and SA2. Both isoform was quantified using isoform specific primers by qPCR using Sybergreen (KAPA 2X qPCR mix) in a Bio-Rad CFX real-time PCR machine as per manufacturer's instructions. Analyses were performed in triplicates and student t-test was used to determine significance. Sequences of minigene constructs and DNA oligos are provided in Supplementary Methods.

### Western blotting

For western blotting, cells were lysed in 1× RIPA buffer (10 mM Tris–Cl (pH 8.0), 1 mM EDTA, 0.5 mM EGTA, 1% (v/v) Triton X-100, 0.1% (w/v) sodium deoxycholate) supplemented with 1× complete mini EDTA protease inhibitor cocktail (Roche) on ice for 15 min. Equivalent amount of total cell lysate was loaded on 10-well precast gels (BioRad). Proteins were resolved for 1 h at 27°C at constant 100 V. Proteins were then transferred to a methanol preconditioned PVDF membrane on a semi-dry apparatus for 30 min at 27°C at constant 16V. Immunoblots were performed using the indicated antibodies and visualized using Pierce enhanced chemoluminiscence (ECL) substrate (Life Technologies). Antibodies and blotting conditions used are detailed in Supplementary Methods.

### Statistics

For next-generation sequencing, single minigene libraries were sequenced per sample. Minigene splice assays for individual minigenes were performed in triplicates. Student's *t*-test was used to determine *P*-value where indicated.

## RESULTS

### SF3B1^K700E^ utilizes upstream 3′ splice sites at higher frequency than SF3B1^WT^

**Figure 1.**
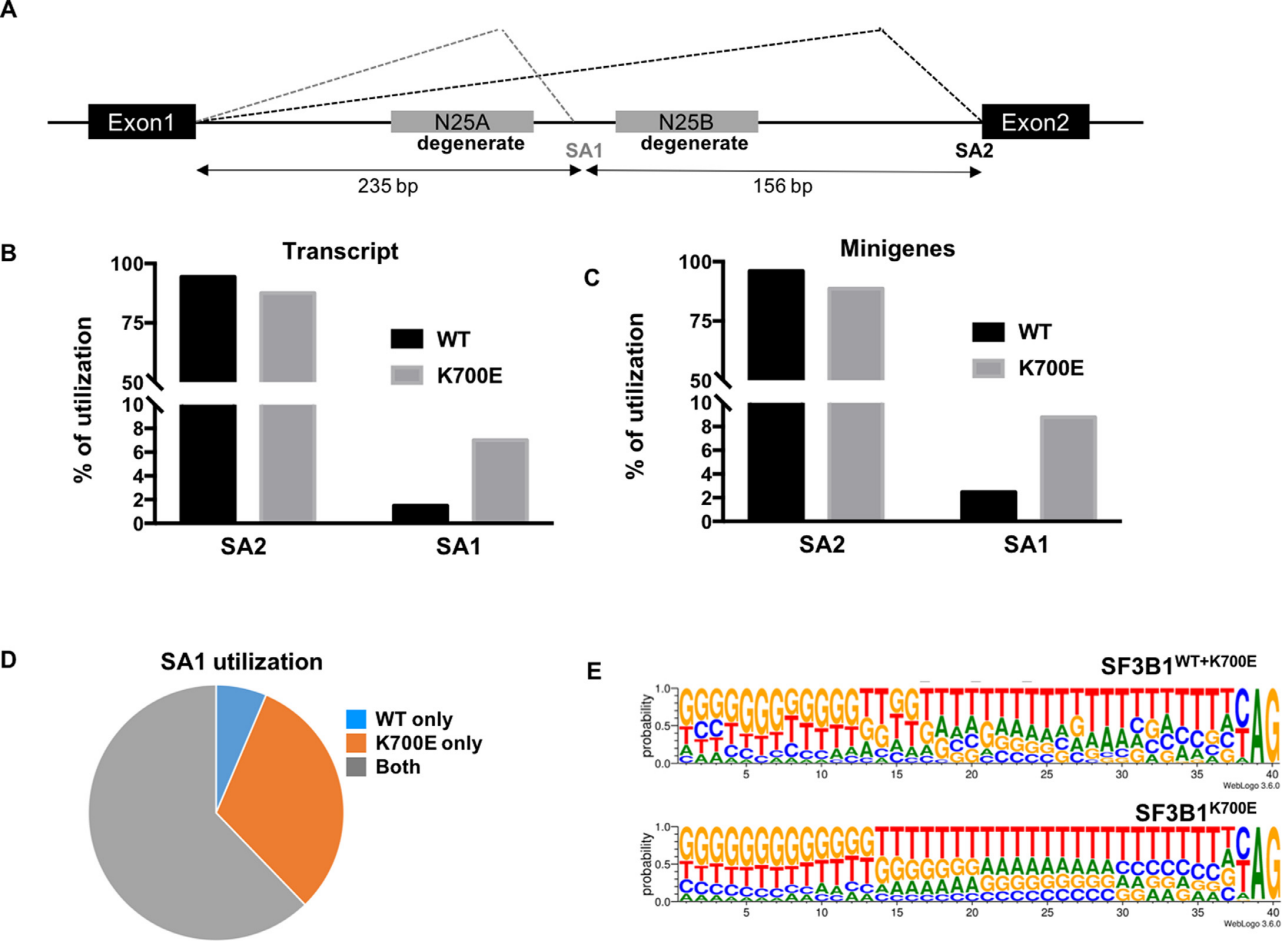
Degenerate minigene library and variable use of 3′SS by SF3B1^WT^ and SF3B1^K700E^. (**A**) Scheme of 3′ minigene library. The two major splice sites are shown (SA1, and SA2) along with two degenerate regions each of 25bp length (N25A and N25B). Distance between the splice sites are indicated in base pairs (BP). (**B**) Variable use of SA1 and SA2 by SF3B1^WT^ and SF3B1^K700E^ cells (total transcript). (**C**) Variable use of SA1and SA2 by SF3B1^WT^ and SF3B1^K700E^ cells (minigene). (**D**) Distribution of 259,187 minigenes that utilize SA1 between three groups (SF3B1^WT^, SF3B1^K700E^ or shared by both SF3B1^WT^ and SF3B1^K700E^. (**E**) Upstream motifs from novel 3′SS (located in degenerate regions) in minigenes spliced by both SF3B1^WT^ and SF3B1^K700E^ (top panel) and exclusively by SF3B1^K700E^ (bottom panel).

We implemented a massively parallel reporter assay (MPRA) using a minigene library with variable 3′SS (23) to determine differences in splice site competition between SF3B1^WT^ and SF3B1^K700E^. This 3′SS minigene library has two 3′ splice sites (termed SA1 and SA2), of which SA1 is framed by two stretches of degenerate nucleotides, each 25 bp long (25A and 25B) as shown in Figure 1A. As shown previously, SA2 is the predominant splice acceptor for most minigenes in this library and SA1 serves as a less frequently used splice acceptor. SA1 selection is strongly influenced by both upstream (25A) and downstream (25B) sequences. We postulated that comparing patterns of degenerate sequence in those minigenes that utilize SA1 in SF3B1WT and SF3B1^K700E^ would give clues to determinants of differential splice site selection.

We first used the human embryonic kidney cell line, HEK293T, and performed co-transfections of SF3B1 and the 3′SS library. Because SF3B1 mutations are hemizygous, it is important to limit expression of plasmid-encoded SF3B1 to physiologic levels. Optimal concentration of SF3B1-encoding plasmid was determined by western blotting (Supplementary Figure S1). Total RNA was isolated at 48 hours after co-transfection, Illumina-compatible cDNA libraries were prepared with minigene specific primers and paired end sequencing was performed (detailed in Materials and Methods). Transcripts were matched to minigenes using unique 20 bp barcodes in the read 2 (R2) of paired end reads. The minigene plasmid library contained over 3.4 million unique components. Transcripts noted to be present in both SF3B1^WT^ and SF3B1^K700E^ datasets at a minimum representation of five reads were used for further comparative analysis (28.9 and 21.8 million transcripts respectively distributed over approximately 700 000 minigenes).

Several groups including ours have previously reported the utilization of novel or ‘cryptic’ 3′SS in SF3B1-mutant cells (11,9,10). These cryptic splice sites are typically found 15–30 bp upstream of their canonical counterparts. We first determined the relative usage of the predominant splice site (SA2), and the alternate, less frequently used splice site SA1. As shown in Figure 1B, both SF3B1^WT^ and SF3B1^K700E^ cells used SA2 as their predominant splice site. However, SF3B1^K700E^ cells used SA1 4.7-fold more often compared to SF3B1^WT^ cells. Other less frequently used splice acceptors within the intron were also noted to be increased in SF3B1^K700E^ compared to SF3B1^WT^ (data not shown). This increase came at the expense of the predominant canonical site SA2. When analysis was done at the level of individual minigenes, a similar trend was noticed (SF3B1^K700E^ utilized SA1 at higher ratios compared to SF3B1^WT^, Figure 1C). Among all the minigenes that utilized SA1 as splice acceptor (total 259 187), 62% were utilized both by SF3B1^WT^ and SF3B1^K700E^, 32% exclusively by SF3B1^K700E^ cells and 6% exclusively by SF3B1^WT^ (Figure 1D). Thus, SF3B1^K700E^ is approximately five times more likely to use SA1 when compared to SF3B1^WT^.

To determine how primary sequence motifs may influence novel 3′SS selection by SF3B1^K700E^, we compared sequence motifs upstream of novel 3′SS in the degenerate regions (N25A or N25B, Figure 1E). We excluded SA1and SA2 in this analysis given that the immediate upstream regions of these splice acceptors are invariant. The sequences upstream of these novel 3′SS were similar in nucleotide distribution among minigenes utilized exclusively by SF3B1^K700E^ or those common to SF3B1^WT^ and SF3B1^K700E^. Previous work using patient samples had suggested the polypyrimidine tract (PPT) upstream of cryptic 3′SS to be weaker and interrupted by adenosines (9–11,15). This difference could be explained by the typical location of cryptic 3′SS in relation to their canonical counterparts (15–30 bp upstream). This position the BP of canonical 3′SS within the PPT of cryptic 3′SS resulting in ‘weak’ PPT interrupted by adenosines. Such an overlap is not present in the upstream sequence of novel 3′SS motifs determined from the minigene library. Taken together, our results show an increased use of non-canonical 3′SS in minigenes by SF3B1^K700E^. However, novel 3′SS used by SF3B1^K700E^ are not distinguished by a weaker PPT or presence of adenosines in their immediately upstream sequence.

### Sequence motifs that serve as enhancers or repressors of splicing are similar in SF3B1^WT^ and SF3B1^K700E^ cells

In previous work using the minigene library, sequence motifs in the degenerate regions either upstream or downstream of SA1 were noted to strongly influence splice site competition (23). The effect of individual sequence motifs on splice site competition was calculated precisely by determining the odds ratio (OR). To better understand how sequence motifs SF3B1^WT^ and SF3B1^K700E^ select 3′SS differently, we used a similar computational algorithm to determine how sequence motifs influence SA1 site selection. First, minigenes that utilized SA1 were identified and the degenerate regions (upstream 25A and downstream region 25B, Figure 1A) were analyzed for the presence or absence of short sequences of 6 nucleotides (6-mers, total 4^6^ = 4096). Odds ratio (OR) for presence of each individual 6-mer in minigenes that utilized SA1 was calculated as described in Materials and Methods. Accordingly, 6-mers with highest OR are associated with the highest probability of being present in minigenes that utilize SA1. Conversely, 6-mers with lowest OR are associated with lower chance of SA1 usage and thus act as splicing repressors.

**Figure 2.**
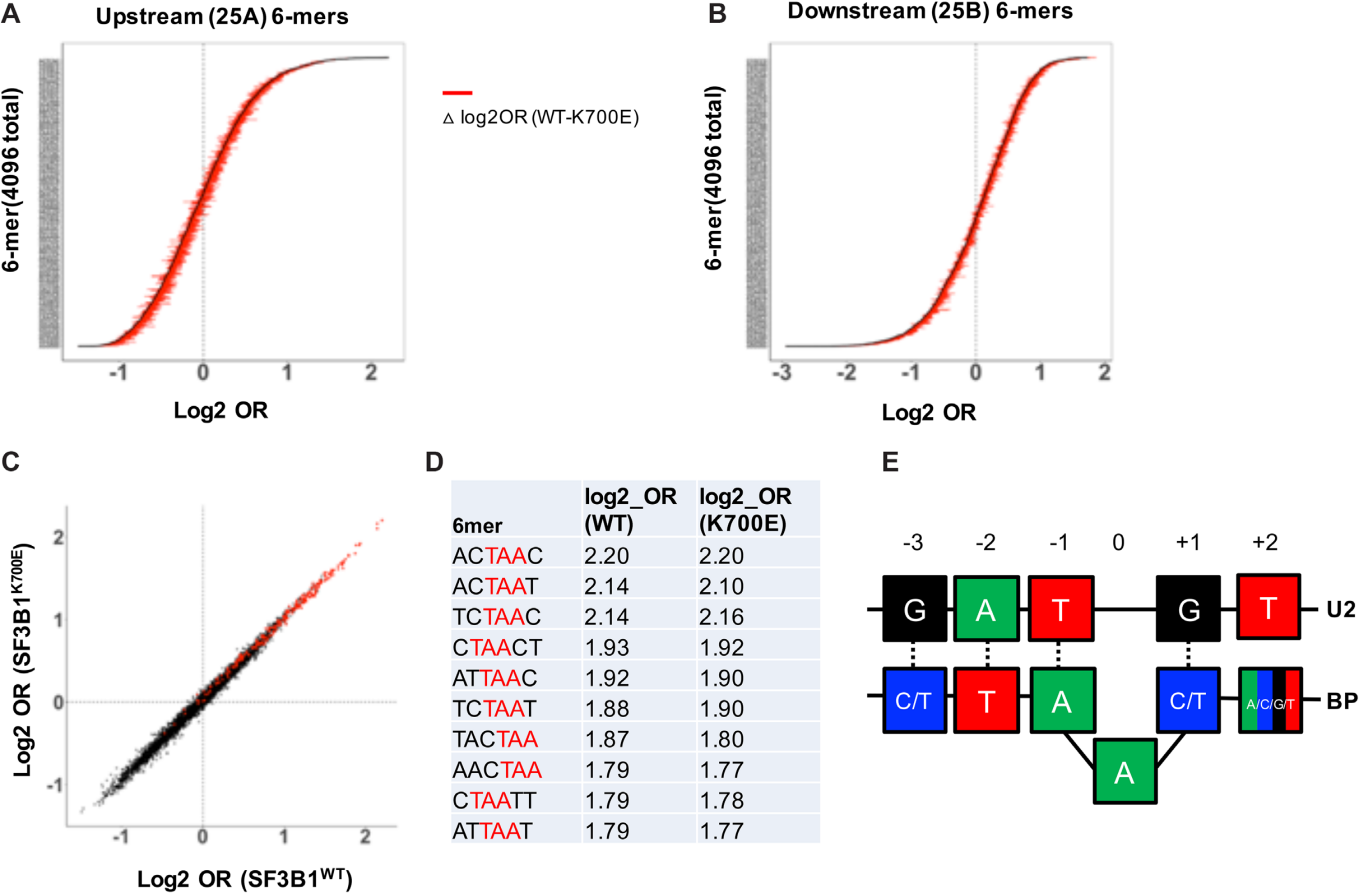
Difference in 6-mer usage by SF3B1^WT^ and SF3B1^K700E^ cells. (**A**) Distribution of log2 odds ratio (OR) of 6-mers in the 25 bp degenerate region upstream of SA1 (25A) for SF3B1^WT^. Red horizontal lines represent difference of log2_OR between SF3B1^WT^ and SF3B1^K700E^ (calculated as Δlog2_OR) for respective 6-mer. (**B**) Distribution of log2_OR of 6-mers in the 25bp degenerate region downstream of SA1 (25bp), plotted similar to Figure 2A. (**C**) log2_OR of SF3B1^WT^ (x-axis) and SF3B1^K700E^ (y-axis) for 4096 6-mers. Red dots indicate those with TAA motif. (**D**) 6-mers with highest log2_OR in 25A region. Trinucleotide TAA is highlighted in red. (**E**) Canonical BP sequence (YTAAY) that base-pairs with U2 snRNA as derived from 6-mers with highest log2OR (Figure 2D).

Pattern of odds ratio (OR) for 6-mers in both SF3B1^WT^ and SF3B1^K700E^ (in 25A and 25B regions) were largely similar and overlapping as shown in Figure 2A–C. As previously noted, the strongest upstream enhancers of SA1 usage were noted to contain the putative BP consensus sequence (CU[AG]A[CU]) and specifically the YTAAY motif which can fully base-pair with U2 snRNA (canonical or wobble, Figure 2D and E). Strong inhibitors of SA1 selection were G-rich sequences likely binding to hnRNP proteins as shown previously (Supplementary File 1) (23). Together, our results show that SF3B1^WT^ and SF3B1^K700E^ spliceosomes do not differ drastically in how use of SA1 is influenced by upstream or downstream sequence motifs.

### SF3B1^WT^ and SF3B1^K700E^ utilize canonical BP similarly, but SF3B1^K700E^ utilizes non-canonical BP better than SF3B1^WT^

**Figure 3.**
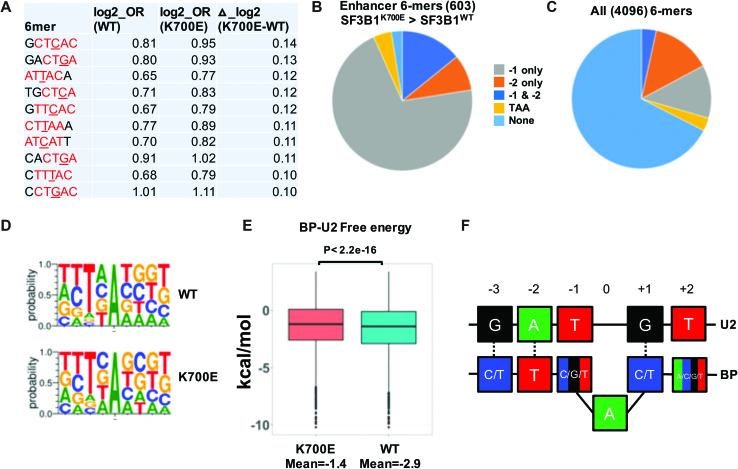
Increased use of non-canonical BP sequences by SF3B1^K700E^. (**A**) Splicing enhancing upstream 6-mer sequences (OR >1.5) with highest difference between SF3B1^WT^ and SF3B1^K700E^ (Δ_log2_K700E-WT). −3 to +1 positions relative to BP adenosine is colored red. Non-canonical nucleotides (varying from the canonical YTAAY sequence) at −1 position are underlined. (**B**) Distribution of 6-mers that enhance splicing (OR>1.5) more in SF3B1^K700E^ compared to SF3B1^WT^. Motifs are divided to those that contain trinucleotide TAA, −1 position variants, −2 position variants, Variants at both −1 and −2 and those have none of the above. A total of 603 6-mers met criteria for this analysis. (**C**) Distribution of all 4096 6-mers to the same categories as described for Figure 5B. (**D**) Sequence logo of BP sequences predicted by LabRanchoR for minigenes using SA1 with SF3B1 wild-type (top panel) and SF3B1^K700E^ (bottom panel). (**E**) Free energy estimate of U2-BP basepairing predicted by LabRanchoR for minigenes that utilize SA1 in SF3B1^WT^ and SF3B1^K700E^. *P* value calculated by wilcox test in the R-package. (**F**) Schematic showing modified base-pairing of BP-U2 snRNA (at −1 position) in non-canonical BP better utilized by SF3B1^K700E^ compared to SF3B1^WT^.

The preponderance of YTAAY motifs among splicing enhancers in the N25A region shows that sequence motifs that fully base pair with U2 snRNA around the BP adenosine are favored when available. Importantly, there is little difference in OR of such canonical BP-containing 6-mers between SF3B1^WT^ and SF3B1^K700E^ datasets. This finding suggests that strong canonical BP sequences are utilized equally between SF3B1^WT^ and SF3B1^K700E^ and hence unlikely to account for their differential utilization of novel 3′SS. We thus considered the possibility that differences in BP utilization may be only reflected in utilization of ‘non-canonical’ BP that do not contain the YTAAY motifs. Human BP are known to be highly degenerate with variations to the canonical BP sequence that allow substitutions at multiple positions around the nucleophilic base (16,18,19). We hypothesized that a change in BP specificity may not be reflected in the use of strong canonical YTAAY sequences, but in non-canonical BP sequences that allow for mismatches in its base pairing with U2 snRNA. To test this, we first determined those 6-mers that enhanced splicing more in SF3B1^K700E^ when compared to SF3B1^WT^ (Supplementary File 2). We found that 6-mers that enhanced splicing with the highest differential in OR (Δlog2_K700E-WT) were highly enriched for non-canonical BP that differed from the YTAAY, at −1 position (Figure 3A). Furthermore, majority of enhancer 6-mers that perform better in SF3B1^K700E^ (∼85%) contained variants at −1 position (Figure 3B) compared to ∼16% predicted for all 4096 6-mers (Figure 3C). Although substitutions at other positions (−2 and +1) were also noted, their proportion was not different from what was anticipated at random. Our computational results points towards a strong trend to better utilize non-canonical BP by SF3B1^K700E^ when compared to SF3B1^WT^.

### Features of U2-BP base pairing in SF3B1^K700E^

In the absence of detailed annotation of vertebrate BP, deep learning models trained on the limited set of available annotated human BP sequences have been used to predict BP utilization from intronic sequences (24,25). We used one such called LabRanchoR (25) to independently predict BP in the degenerate regions upstream of SA1. LaBRanchoR determines the probability of each base (from a total of 70 bp) pair upstream of a 3′SS to be the nucleophilic base for BP. We compared the sequence motifs of putative BP sequences (9-mers with the fifth base being the nucleophilic base) for minigenes that were spliced by both SF3B1^WT^ and SF3B1^K700E^ (Common_BP), or only by SF3B1^K700E^ (K700E_BP). Our results were in agreement with analysis based on odds ratio (OR) and showed variation from the canonical BP sequences. While the nucleophilic base itself was almost always A, the −2 base was U less often in SF3B1^K700E^ (68.8% versus 79.3% in SF3B1^WT^, Figure 3D and Supplementary Figure S2). Finally, we determined how the energetics of BP region base-pairing with U2 snRNA may differ in SF3B1^K700E^ compared to SF3B1^WT^. Based on the presence of non-canonical nucleotides in −1 position, we hypothesized that SF3B1^K700E^ allows higher energy interactions between U2 snRNA and BP. We determined the predicted binding energy of U2-BP using RNAduplex algorithm (26) and found that interactions of the U2-BP duplex for SF3B1^K700E^ were significantly less stable compared to SF3B1^WT^ (free energy means of −1.40 versus −2.92 kcal/mol respectively with a *P*-value < 2.2e–16, Figure 3E). Taken together, BP prediction of SF3B1^K700E^ appear to support a model of SF3B1^K700E^ BP utilization that accommodates non-canonical nucleotides in the immediate vicinity of the nucleophilic adenosine thereby allowing less stable U2-BP binding (Figure 3F).

**Figure 4.**
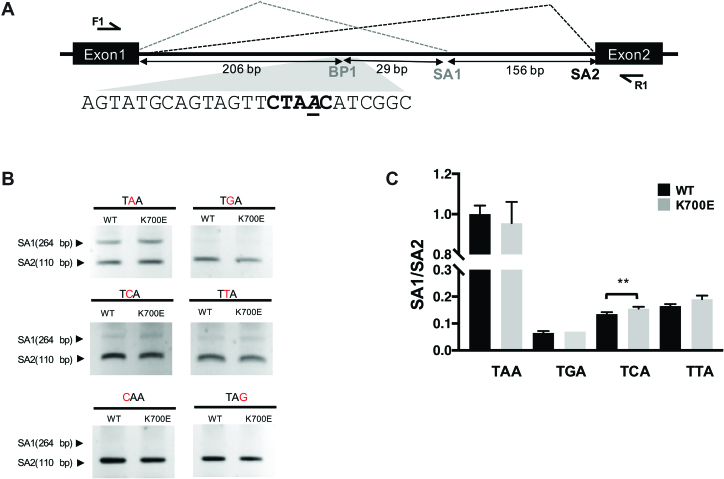
Minigene splicing assay to determine change in isoform ratios of SA1 and SA2 utilization with variation of BP sequence. (**A**) Minigene with canonical BP sequence CTAAC highlighted. BP adenosine is underlined. Two splice sites SA1 and SA2 are marked. Location of primers for amplification of minigene transcript are also shown (arrow). (**B**) Agarose gel electrophoresis of minigenes with different BP substitutions. Amplification of isoform that utilizes SA1 produces a 264 bp product while amplification of SA2 results in a 110 bp product. Top and middle panels show control vector (TAA) and variations at −1 position (TGA, TCA, TTA). Lower panel shows additional variants (C at −2 position or replacing the BP adenosine by G). (**C**) Quantitative PCR of isoforms that utilize SA1 or SA2 (using junction-spanning isoform-specific primers). Ratio of SA1 use to SA2 use was calculated for each minigene, the SF3B1^WT^ value was normalized to 1. Error bars represent SEM of three replicates. ** represents *P* value < 0.02 by Student's *t*-test.

To confirm the above computational model, we performed minigene splicing assays using a series of minigene constructs. These minigenes were derived from the 3′SS library with the same two exons of citrine with an intervening intron as well a 3′UTR (Figure 4A). Single nucleotide variant minigenes (at −1, −2 or 0 positions) were generated through site-directed mutagenesis. Minigenes were then co-transfected with SF3B1^WT^ or SF3B1^K700E^ in HEK293T cells and total RNA extracted at 48 hours, reverse-transcribed and analyzed by PCR (gel electrophoresis and quantitative PCR). A small but significant change in SA1 use was noted in SF3B1^K700E^ when compared to SF3B1^WT^ in −1 variant minigene (Figure 4B and C) confirming our computation-derived results. Changing the −2 position nucleotide to C or the nucleophilic (0 position) nucleotide to G resulted in abrogation of SA1 use. Splicing assays thus confirm an improvement in the use of non-canonical (–1 position variant) BP in mutant SF3B1 when compared to SF3B1^WT^.

We also implemented the LabRanchoR algorithm to patient samples by comparing the putative BP in upstream sequences of cryptic and canonical 3′SS that we previously identified from myelodysplastic syndrome (MDS) and chronic lymphocytic leukemia (CLL) (11). However, we could not find a difference in U2-BP binding energy between cryptic and canonical 3′SS pairs. (Supplementary Figure S3 and Supplementary File 3). We attribute this to two key differences between our minigene approach and cryptic/canonical 3′SS comparisons: (i) total cryptic/canonical 3′SS available for comparison are only in the several hundreds and (ii) over half of the cryptic 3′SS lie in close proximity of their canonical counterparts (<50 bp) resulting in significant overlap in their upstream sequences. Our results underscore the utility of our approach based on MPRA over analysis restricted to patient samples.

### Generation of isogenic *Sf3b1*-mutant mouse embryonic stem cells (mESC) and confirmation of changes to BP usage

**Figure 5.**
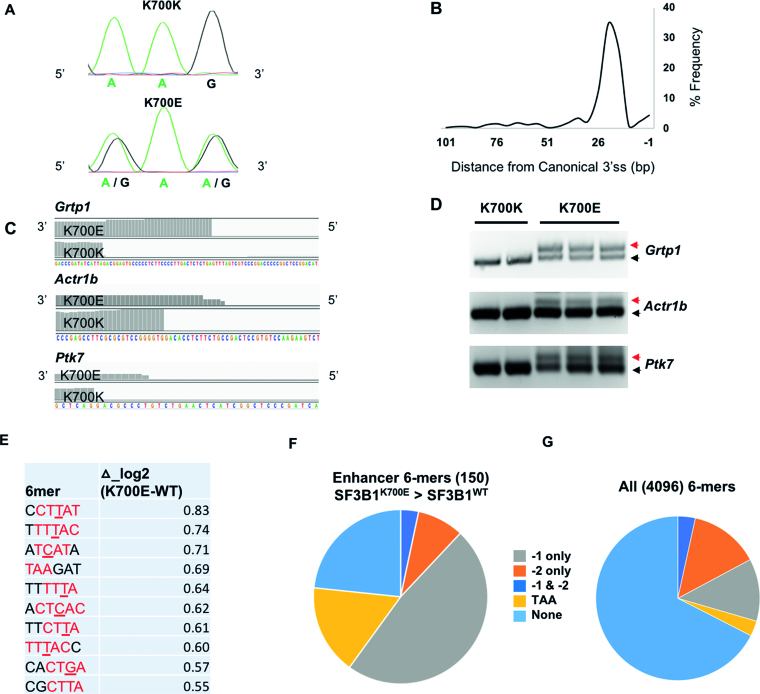
Generation of isogenic Sf3b1-mutant mouse embryonic stem cells (mESC) and confirmation of changes to BP usage. (**A**) Analysis of SF3B1 mRNA expression from edited alleles by Sanger sequencing of cDNA isolated from SF3B1^K700K^ and SF3B1^K700E^ mESC. (**B**) Distribution of the distances of cryptic 3′SS in relation to their canonical 3′SS are shown for Sf3b1^K700E^ mESC, as determined by RNA-seq. (**C**) Cryptic 3′SS in three candidate genes identified through comparative anlaysis of RNA-Seq from SF3B1^K700K^ and SF3B1^K700E^ mESC clones. (**D**) Confirmation of cryptic 3′SS in Figure 5C by reverse transcription, PCR and gel electrophoresis. Red and black arrows represent isoforms associated with cryptic 3′SS and canonical 3′SS selection, respectively. (**E**) Splicing enhancing upstream 6-mer sequences (OR >1.5) in mESC datasets with highest difference between SF3B1^WT^ and SF3B1^K700E^ (Δ_log2_K700E-WT), as shown in Figure 3A. −3 to +1 positions relative to BP adenosine is colored red. Non-canonical nucleotides (varying from the canonical YTAAY sequence) at −1 position are underlined. (**F**) Distribution of 6-mers that enhance splicing (OR > 1.5) more in SF3B1^K700E^ compared to SF3B1^WT^ in mESC datasets (total of 150 6-mers met criteria). Motifs are divided to those that contain trinucleotide TAA, −1 position variants, −2 position variants, Variants at both −1 and −2 and those have none of the above. (**G**) Expected distribution of all 4096 6-mers to the same categories as described for 5F.

To confirm the effect of SF3B1 mutations on splice-site usage and splicing outcomes in an experimental system that more faithfully reproduces genetics of disease associated with mutant SF3B1, we generated isogenic murine embryonic stem cells (mESC) expressing the Sf3b1^K700E^ mutation from its endogenous locus using CRISPR-Cas9 genome editing and homology directed repair (HDR). We selected a guide-RNA (gRNA) that directed Cas9 to exon 15 of *Sf3b1* close to the K700 position where it introduced bi-allelic double-stranded breaks (DSBs), as confirmed by the SURVEYOR mutation detection assay (Supplementary Figure S5B). We first attempted single allele editing using the Cas9/gRNA complex along with a HDR vector that contained a neomycin (Neo) resistance gene flanked by sequences homologous to the genomic region surrounding and containing the K700E (AAA→GAA) mutation. Although Cas9 introduced bi-allelic DSBs, we consistently found that only one allele underwent HDR to incorporate the repair vector containing K700E while the other allele underwent non-homologous end joining (NHEJ) that introduced insertions and deletions (INDELs). In order to faithfully replicate disease genetics in which SF3B1 hotspot mutations are almost always hemizygous, we developed a dual HDR vector strategy to ensure that while one allele was edited by the introduction of K700E (AAA→GAA) mutation, the other allele did not contain INDELs (Supplementary Figure S4A and B). Using this strategy, we generated mutant *Sf3b1* K700K/K700E mESC (referred as SF3B1^K700E^) and control *Sf3b1* wild-type (K700K/K) mESC (referred as SF3B1^K700K^). Integration of the repair vectors was observed at similar frequencies for both K700K and K700E (Supplementary Figure S6B) and was confirmed by PCR (Supplementary Figure S5A) and Sanger sequencing of genomic DNA (Supplementary Figure S6C) as well as cDNA (Figure 5A). We confirmed the expression of full-length *Sf3b1* at the mRNA (Supplementary Figure S6D) and protein levels (Supplementary Figure S6A) in both SF3B1^K700E^ and SF3B1^K700K^ cells. We further characterized our gene targeting strategy using the GUIDE-Seq methodology (27), which demonstrated only a single off-target editing event induced by our gRNA involving a coding gene (*Fam131b*) with poorly characterized function (Supplementary Figure S5B and C).

We then confirmed aberrant 3′SS usage induced by *Sf3b1*^K700E^ mutation in mESC by high-depth paired end RNA-Seq as previously described (11). 658 cryptic 3′SS were found in *Sf3b1*^K700E^ (Supplementary File 4). Importantly, they were clustered 15–30 bp upstream of their canonical counterparts (Figure 5B), similar to that reported in patient samples (9–11,15). Three cryptic 3′SS thus identified were confirmed through RT-PCR (Figure 5C and D). Analysis of alternative splicing events using the rMATS algorithm also revealed a splicing pattern similar to that seen in patient samples (Supplementary Figure S7A and B).

Having confirmed similar splicing outcomes of SF3B1^K700E^ mutation between human and mouse cells, we used the mESC to confirm the minigene transfection results as described for 293T cells. Minigene libraries were transiently transfected and cDNA libraries prepared and sequenced as for 293T cells. Comparable to our results with SF3B1^K700E^-overexpressing human cells, SF3B1^K700E^ mESC showed an increase in SA1 use at the expense of SA2 (Supplementary Figure S8A and B). We then looked for evidence of change in BP usage by 6-mer frequencies in SA1-utilizing minigenes. Strongest upstream enhancers of SA1 selection were 6-mers that contained the YTAAY motif (Supplementary File 5). Importantly, splicing enhancing 6-mers with highest Δ_log2-OR were variants of the canonical YTAAY at −1 position in 9 of 10 cases (Figure 5E). Nearly half of such 6-mers with positive Δ_log2-OR values contained −1 variants of the canonical YTAAY motif (similar to results of experiments performed in 293T cells, Figure 5F and G). In summary, these data using isogenic cell lines confirm the change in BP usage by mutant SF3B1.

## DISCUSSION

In this study, we performed a MPRA using a minigene library with degenerate sequences surrounding 3′SS to define nature of BP utilization in SF3B1-mutant cancers. Several studies using patient samples, animal models and cell lines have all shown mutant SF3B1 expression to be associated with novel or cryptic 3′SS (9–12,15,28). Based on SF3B1’s critical role in stabilizing U2 snRNA-BP interaction, it has been speculated that a change in BP is the primary driver of this change in 3′SS. Analyzing a limited number of cryptic 3′SS, Alsafadi *et al*. proposed a model wherein SF3B1^K700E^ preferentially used novel BP motifs with stronger binding to U2 snRNA (15). A separate, limited genome-wide analysis of BP utilization in an isogenic SF3B1^K700E^ cell line did not reveal patterns unique to mutant spliceosomes (16). Defining BP in an unbiased fashion has proven difficult due to (1) the degenerate nature of BP sequences in vertebrates making their informatics prediction difficult; (2) technical challenges in isolating BP-containing intronic lariat sequences; and (3) low total number of cryptic 3′SS from patient samples, typically a few hundred, limiting robust statistical analysis. Additionally, cells that express mutant-SF3B1 alone (without the wild-type protein) are non-viable making it difficult to isolate the effects of mutant-spliceosome from that of the wild-type spliceosome at the cellular level.

Our experimental approach allowed us to calculate the odds ratio (OR) of individual sequence motifs to influence selection of 3′SS. Several features of 3′splice site selection were evident through this analysis: (1) similar to patient samples, SF3B1^K700E^ utilizes novel 3′SS in the minigene library when compared to SF3B1^WT^; (2) both SF3B1^K700E^ and SF3B1^WT^ are similar in their utilization of strong promoters or inhibitor sequences upstream or downstream of 3′SS; (3) upstream sequence motifs containing a consensus motif YTAAY (predicted to base-pair with U2 snRNA) are strongest enhancers of 3′SS selection in both SF3B1^WT^ and SF3B1^K700E^ and (4) SF3B1^K700E^ utilizes motifs with non-canonical BP sequences (specifically those which vary at position −1 relative to BP adenosine) more often than SF3B1^WT^. This finding of altered BP preference by mutant SF3B1 was corroborated by minigene assays as well as BP predictions using an independent, deep learning network (LabRanchoR). Together, these results point to a key difference between SF3B1^WT^ and SF3B1^K700E^ with respect to splice site utilization. While canonical BP (with strong base-pairing with U2 snRNA) are favored by SF3B1^WT^ and SF3B1^K700E^, non-canonical variants are utilized by SF3B1^K700E^ at higher rates compared to SF3B1^WT^. The effect is however relatively modest and in line with the overall modest change in splicing events seen across multiple models of mutant SF3B1. After performing an initial analysis in a gene dose-optimized overexpression model, we generated isogenic mESC cell lines that accurately model the single-allele nature of disease-associated SF3B1 mutations in patient samples. These studies in non-transformed cells confirmed our findings from the over-expression model. The nature of BP utilization by cancer-associated SF3B1 mutations was explored recently in two studies in yeast (29, 30), which determined splicing fidelity of non-canonical BP by mutant proteins. Some of the mutants including the P369E mutation (corresponding to human K700E) increased the use of such non-canonical BP while others decreased it. Differences in yeast and human cells, including the requirement for additional factors in recognizing the highly degenerate human BP, may be responsible for such dissimilarities in BP utilization by mutant SF3B1 in these experimental systems.

**Figure 6.**
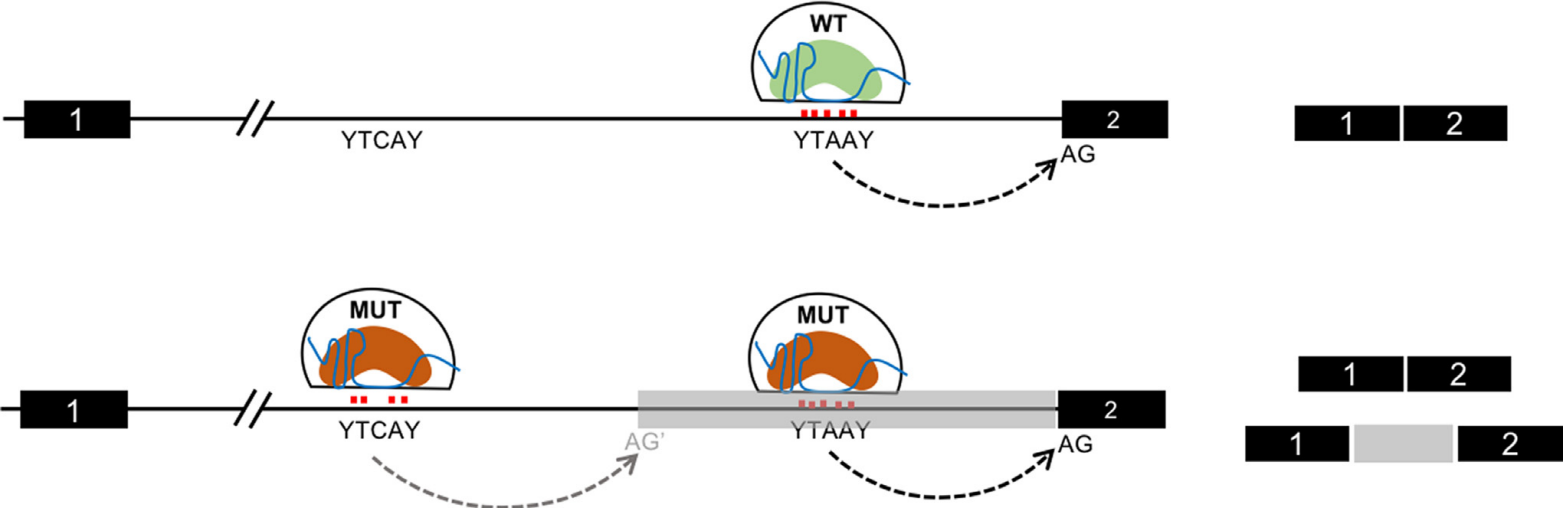
Model of BP utilization by wild-type and mutant SF3B1. Wild-type spliceosomes (upper panel) recognize and utilize canonical BP with stable BP-U2 interactions, which lead to use of canonical 3′SS only. Mutant spliceosomes (lower panel) utilize canonical BP preferentially but are also able to utilize non-canonical BP sequence with less favorable U2-BP energetics, leading to novel splice isoforms.

Based on our results, we propose a model of BP utilization wherein weaker BP-U2 snRNA interactions can be stabilized preferentially by SF3B1^K700E^. This allows for the use of novel 3′SS when potential 3′SS are located downstream of such novel BP (Figure 6). Importantly, the model allows for occasional use of new BP by SF3B1^K700E^, but stronger canonical BP if present will be still favored. This may explain why isoforms with cryptic 3′SS typically constitute only a small proportion of the transcript (low PSI or percentage spliced in) and not close to 50% as would be expected if change in SF3B1^K700E^’s BP was absolute. Other reasons for low PSI include variable allele frequency (VAF) in clinical samples (31) and nonsense mediated decay (NMD) of aberrantly spliced transcripts (10), neither of which are applicable to the minigene model (all cells express mutant SF3B1 and NMD cannot be determined due to the single-intron nature of the minigene). Our model is based on a minigene library with two potential splice acceptors located 156 bp apart, hence it may not be extrapolated to scenarios where competing splice sites are much closer or much further apart. Our model contrasts with a model proposed by Alsafadi *et al.*, in which mutant SF3B1 favored BP with stronger U2 base-pairing (15). This model was however based on analysis of a few selected BP/ 3′SS sequences and not an unbiased genome-wide analysis.

We speculate that several potential mechanisms could allow higher energy interactions between U2 and BP in mutant-SF3B1 cells. Recent work in the yeast model has proposed a disrupted interaction between mutant-SF3B1 and Prp5 (30), a helicase that proofreads the BP. The role of Prp5 is however not well defined in human splicing. While our results strongly suggest the ability of mutant SF3B1 to induce a change in BP recognition, it is not implicit that a change in BP is necessary or sufficient for novel 3′SS selection. Recent genome-wide mapping of human BP have shown instances of single 3′SS being associated with multiple BP (18,19). Conversely, the same BP may use multiple 3′SS. Structural studies of the spliceosome and SF3B1 have shown that in addition to its interaction with the BP region, the C-terminal HEAT domains also interact with the mRNA itself. Interpreting these studies, Jenkins and Kielkopf suggested that the HEAT-domain mutations of SF3B1 may alter its conformation in a way that reduces the effective minimum distance between BP and 3′SS, allowing use of novel 3′SS (32). Other mechanisms may also be at play: for example, we have reported previously that the secondary structure of nascent pre-mRNA influences 3′SS selection by mutant SF3B1 (11). Taken together, it would appear that altered BP utilization is one of multiple mechanisms that lead to aberrant 3′SS selection in mutant cells.

MPRA based on next-generation sequencing have proven useful in defining rules of transcription, translation and splicing (23,33–36). Our results show their utility to define aberrant biochemistry of neomorphic proteins, especially in instances where transcriptome-wide analysis is not fully informative. This minigene approach has some limitations: for instance, the design of the construct (single intron) did not allow us to determine if non-sense mediated decay (NMD) plays a role in altered transcript abundance as has been speculated in some studies of mutant-SF3B1 function (10). Nonetheless, our findings extend the current understanding of BP selection in SF3B1-mutated diseases by demonstrating that mutant SF3B1 can better utilize non-canonical BP sequences when compared to wild-type protein, without losing the ability to use canonical BP sequences, consistent with the notion that recurrent HEAT domain mutations convey gain-of-function pathology.

## DATA AVAILABILITY

All next generation sequencing files generated as part of this project have been deposited with the NCBI Gene Expression Omnibus (GEO) under accession number GSE115547.

## Supplementary Material

Supplementary Data
