# Supplementary material for: Degenerate minigene library analysis enables identification of altered branch point utilization by mutant splicing factor 3B1 (SF3B1)
Source: Nucleic Acids Res. 2018 Nov 20;47(2):970–80. doi: 10.1093/nar/gky1161 (PMC6344872; doi:10.1093/nar/gky1161)
Supplement: Supplementary Data [file gky1161_supplemental_files.zip › Supplementary_Gupta.pdf]

SUPPLEMENTARY FIGURES

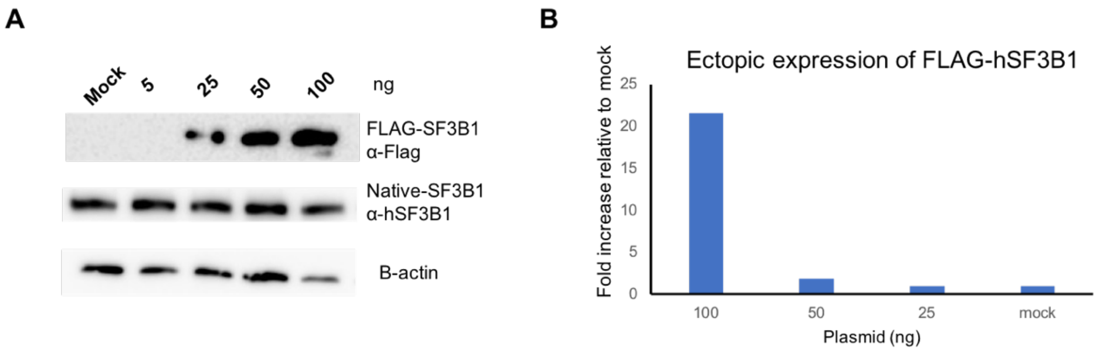

Supplementary Figure 1. Optimization of ectopic SF3B1 expression in HEK293T cells.

A) Western blot analysis of HEK293T cells transiently transfected cells in 24 well plate with indicated amount of plasmid (pCDNA3.1-FLAG). Blot was re-probed with native human SF3B1 to determine total SF3B1. B-actin was used for normalization.
B) Quantitation of total SF3B1 relative to mock-transfected controls. Western blot quantification of total SF3B1 relative to mock. Cells transfected with 50 ng of plasmid showed ~ 1.9 fold expression compared to mock controls.

WT

POSITION	-4	-3	-2	-1	0	+1	+2	+3	+4
A	29.33	12.91	8.96	31.38	99.81	20.38	19.30	19.55	13.24
C	14.56	33.87	5.48	23.90	0.18	26.71	29.53	22.56	20.82
G	25.49	17.31	6.27	23.16	0.01	22.84	30.90	29.80	28.40
T	30.61	35.91	79.30	21.56	0.00	30.07	20.27	28.10	37.55

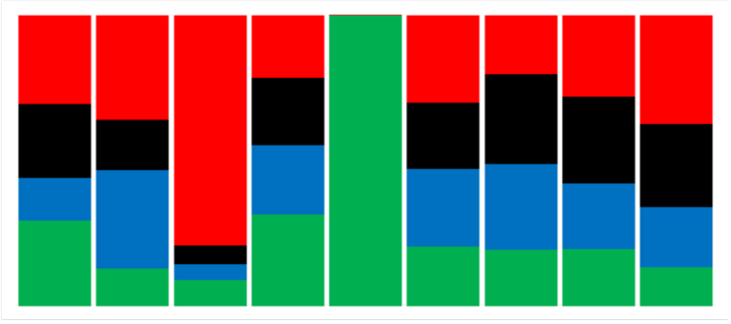

K700E

POSITION	-4	-3	-2	-1	0	+1	+2	+3	+4
A	26.14	14.17	11.48	21.94	99.61	21.21	18.90	18.54	12.26
C	16.64	28.87	9.18	28.17	0.36	21.58	33.08	20.60	20.27
G	26.55	20.45	10.52	26.19	0.03	31.52	29.97	31.10	30.52
T	30.67	36.51	68.82	23.70	0.01	25.69	18.05	29.75	36.94

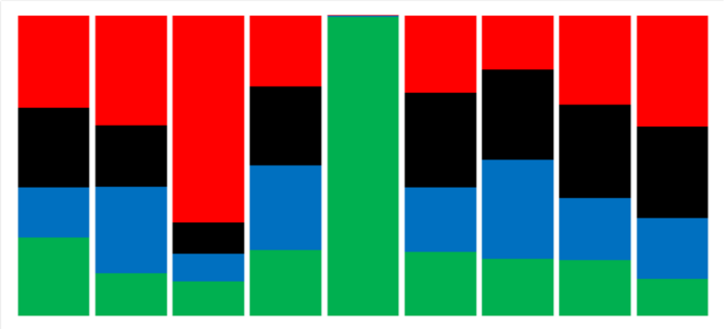

Supplementary Figure 2. Distribution of nucleotides at BP.

Distribution of the 4 nucleotides (shown as percentage of total) at nucleotide positions relative to the nucleophilic base which is denoted as position 0, as predicted by LabRanchoR and shown in Figure 3. Top panel shows minigenes in SF3B1-WT and bottom panel shows minigenes in SF3B1-K700E.

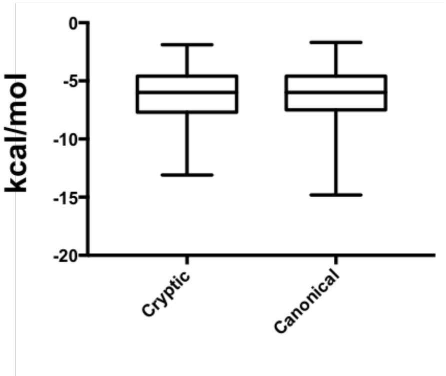

Supplementary Figure 3. Free energy of U2-BP in patient samples

Box plot comparison of free energy predictions (RNAhybrid) of putative BP predicted by labRanchoR in the 70 bp sequence upstream of 414 cryptic and canonical 3'SS pairs identified from myelodysplastic syndromes (MDS). Differences were not statistically significant.

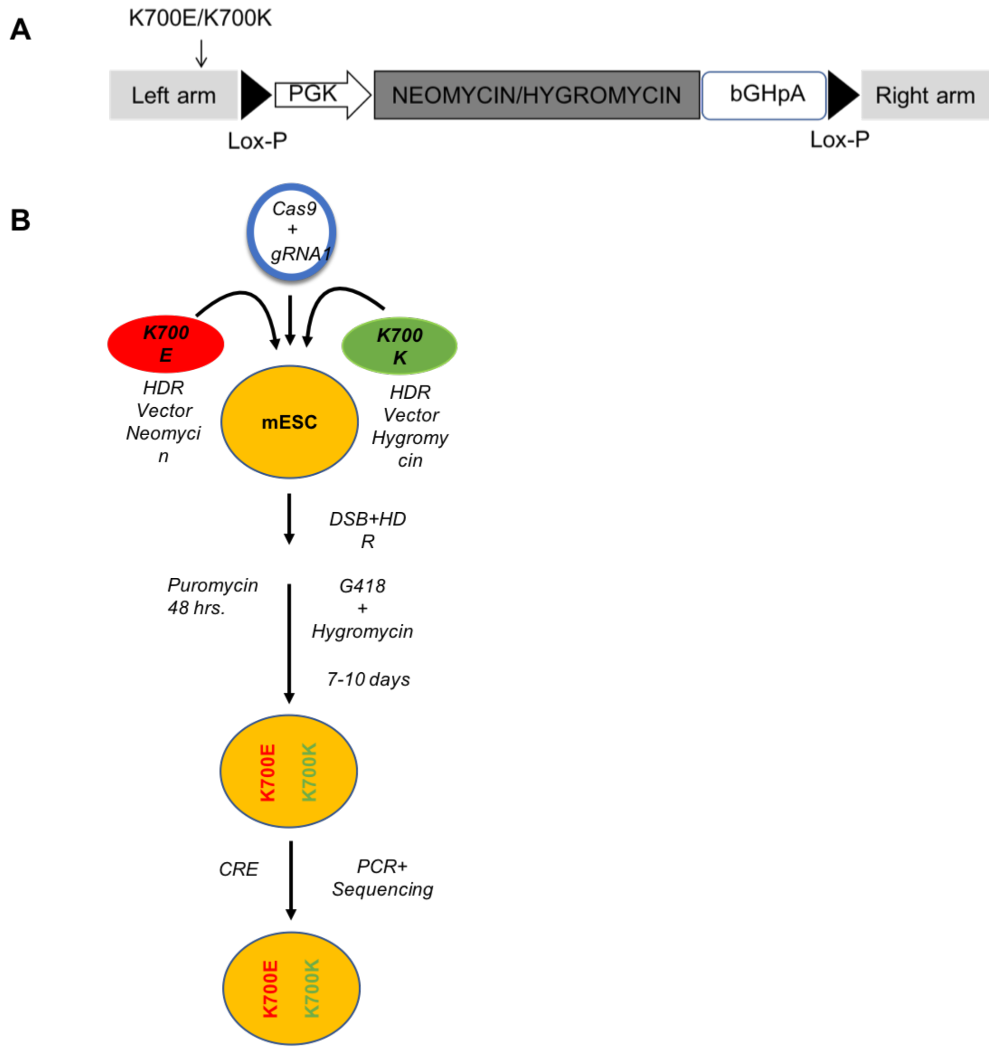

Supplementary Figure 4. Strategy to generate Sf3b1-mutant mESC.

(A) Schematic of HDR vector generated for generation of isogenic cells lines. The construct was constructed using the pL452 vector (Addgene #19178)(1). It consists of a left homology arm (that contains a K700E or K700K mutation), human PGK promoter driving expression of Neomycin or Hygromycin resistance genes, bovine growth hormone polyA (bGHpA) and a right homology arm. Loxp sites were placed as indicated to excise out the PGK-Neo/Hygro/bGHpA cassette after recombination was confirmed.

(B) Targeting of mESC to introduce hemizygous Sf3b1^{K700E} mutation using CRISPR-Cas9 and a dual vector HDR strategy. The Cas9-gRNA complex was introduced into mESC in conjunction with two HDR vectors. One vector contained the neomycin resistance gene flanked by homology arms containing the K700E mutation, while the other vector contained a hygromycin resistance gene containing a synonymous K700K mutation in the homology arm. Similarly, a control Sf3b1 K700K/K700K mESC line was generated.

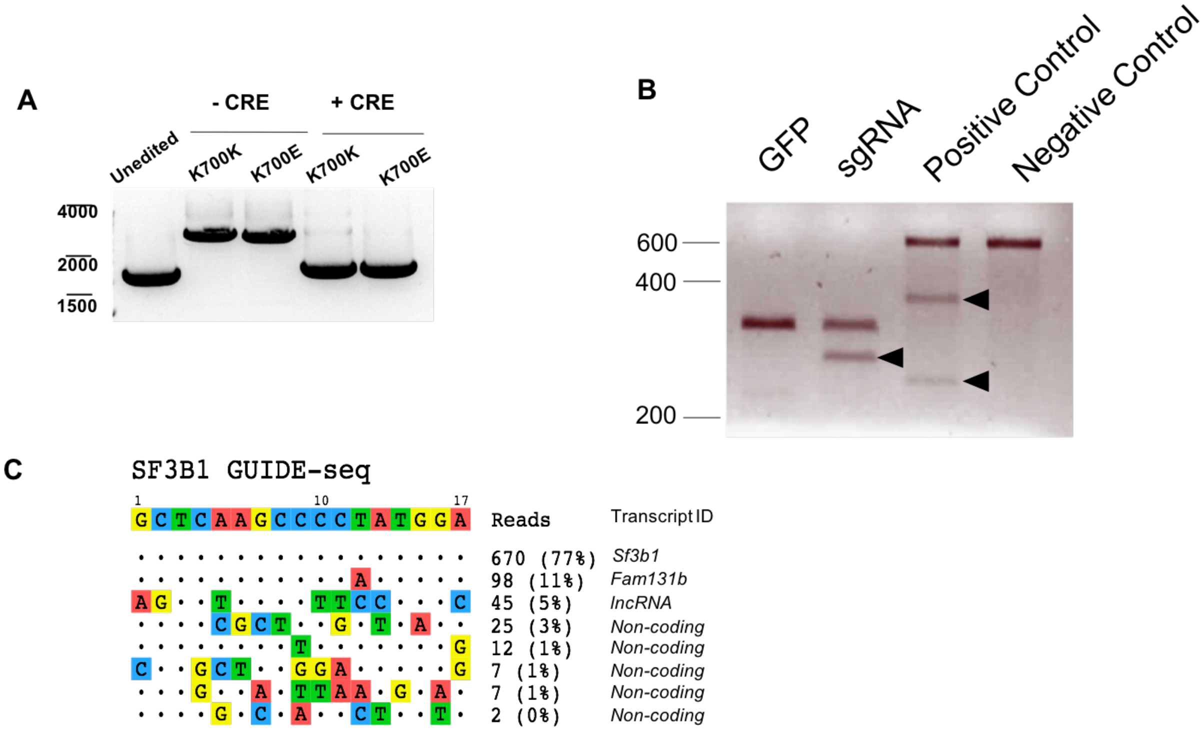

Supplementary Figure 5. Characterization of isogenic Sf3b1-mutant mESC.

A) PCR analysis of genomic DNA isolated from unedited, Sf3b1^{K700K} and Sf3b1^{K700E} mESC pre and post Cre excision of the antibiotic resistance genes.
B) SURVEYOR mutation detection assay to assess double-stranded breaks (DSBs) introduced by the Cas9-gRNA complex. Arrowheads, indicative of mismatches resulting from INDELs, point to cleavage products of PCR fragments amplified from genomic DNA of mESC transfected with Cas9-gRNA.
C) GUIDE-seq was performed with mESC transfected with the Cas9-gRNA. mESC transfected with Cas9 alone were used as control. The intended target site for the Cas9-gRNA complex is shown in the top line. Cleaved sites identified by GUIDE-seq are shown underneath with matching nucleotides represented by dots and mismatches in highlighted text. GUIDE-seq sequencing read counts and their frequencies for each site are shown on the right.

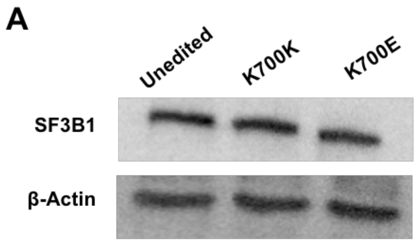

B

	Recombined Alleles	
HDR	1	2
K700K/K	14/54 (25.9%)	20/54 (37%)
K700E/K	13/57 (22.8%)	23/57 (40.3%)

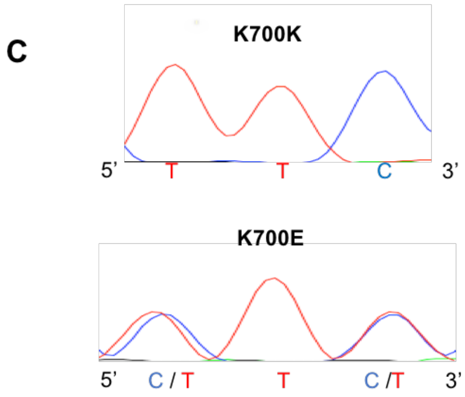

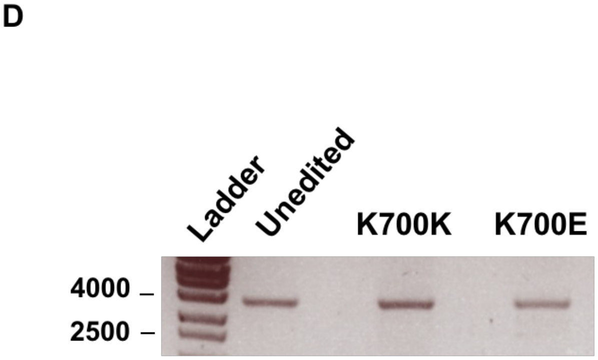

Supplementary Figure 6. Characterization of isogenic Sf3b1-mutant mESC.

A) SF3B1 protein expression by western blot analysis from cell lysates extracted from unedited, Sf3b1^{K700K} and Sf3b1^{K700E} mESC.
B) Frequency of recombination at one or both alleles resulting from the editing of mESC using the dual HDR vector strategy.
C) Confirmation of successful editing by Sanger sequencing of genomic DNA isolated from K700K and K700E mESC.
D) Analysis of the expression of full-length Sf3b1 mRNA in unedited, K700K and K700E mESC by PCR amplification from cDNA.

A

EVENT	DETECTED	ALTERED (p<0.05)
A5'SS	1413	177
A3'SS	2370	352
RI	2065	473
SE	29137	2407
MXE	4650	1534
Total	39635	4943

B

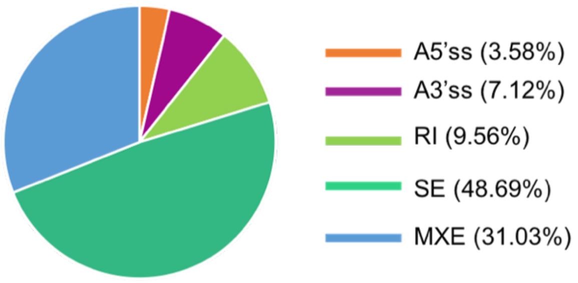

Supplementary Figure 7. Analysis of 3'SS minigene library expressed in Sf3b1^{K700E} and Sf3b1^{K700K} mESC.

(A) Frequency of differential alternative splicing events detected and altered (p<0.05) between K700K and K700E mESC determined using rMATS. Of A5'ss (Alternative 5'ss), A3'ss (Alternative 3'ss), RI (Retained intron), SE (Skipped exon), MXE (Mutually exclusive exons), SE were the most frequent differential alternative splicing events.

(B) Venn diagram showing distribution of the 5 splicing events shown in panel (A).

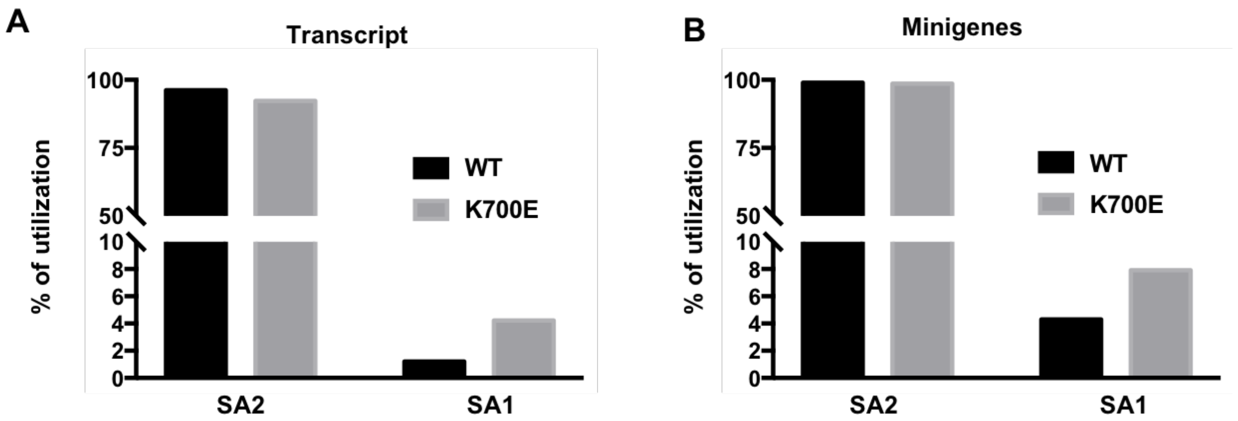

Supplementary Figure 8. Differential use of 3'SS by Sf3b1^{K700E} and Sf3b1^{K700K} mESC.

(A) Differential use of major splice sites at transcript level. Proportion of transcripts using each of the major splice sites is shown.
(B) Differential use of major splice sites at minigene level. Proportion of individual minigenes using each of the major splice sites is shown.

SUPPLEMENTARY FILE LEGEND

Supplementary File 1. Odds Ratio (OR) determined for 6-mers (4096) in 25A region for minigenes that utilize SA1 splice site (HEK293T transfections). Odds and Odds Ratio as well as Log2_OR are shown for each 6-mer as described in Supplementary Methods.

Supplementary File 2. Odds Ratio (OR) of subset of 6-mers (total 603) that promote use of SA1 (OR >1.5) in HEK293T cells. Data derived from the full 4096 6-mers as shown in Supplementary File 1.

Supplementary File 3. LabRanchoR analysis of cryptic 3'SS. Shown are coordinates of cryptic 3'SS, canonical 3'SS, upstream 70 bp sequence, mostly probable BP as predicted by LabRanchoR and predicted energy of U2-BP interaction.

Supplementary File 4. Cryptic 3'SS in Sf3b1^{K700E} mESC.
Shown in columns from left to right are chromosome of cryptic 3'SS, cryptic 3'SS start, cryptic 3'SS end, unique id of each cryptic 3'SS, strand (+ or -) of transcript featuring the cryptic 3'SS and distance from corresponding canonical 3'SS (negative values indicate location 3' of the canonical 3'SS).

Supplementary File 5. Odds Ratio (OR) determined for 6-mers (4096) in 25A region for minigenes that utilize SA1 splice site (mESC transfections). Odds and Odds Ratio as well as Log2_OR are shown for each 6-mer as described in Supplementary Methods**.**

SUPPLEMENTARY METHODS

Total RNA isolation, Reverse transcription and NGS library preparation.

Sequences of DNA Oligos are provided in Supplementary Table 1. Total RNA was extracted prepared using the RNEasy kit (Qiagen), followed by DNAse digestion using Turbo DNase from Ambion as per manufacturer's instructions. Reverse transcription was carried out with MMLV reverse transcriptase (NEB) using oligo dT (Invitrogen). Briefly, approximately 2 ug of total RNA was mixed with 1 ul of 10mM dNTP and 5 um of oligo dT in a total volume of 16 ul, mixture was incubated at 65 degrees for 5 min followed by snap chill on ice for 5 min. 2 ul of 10x RT buffer, 40 U of RNasin (NEB) and 200 U of MMLV-RT was added and incubated at 42 degrees C for one hour followed by inactivation at 90 degrees C for 10 minutes. The quality of cDNA and presence of DNA contamination was checked by regular PCR using primers (F1 and R1) spanning the exons of citrine and was compared to No RT control.

Illumina compatible libraries were generated from cDNA by using two consecutive overlapping primer PCR approach. Briefly, 10 % of cDNA was first PCR amplified with equimolar mixtures of staggered forward primer (F2 to F7) and reverse primer (R2) spanning the single intron of citrine using high fidelity 2X NEB master mix (NEB) as per manufacturer's instructions. Cycling was done on a Bio-Rad C1000 thermal cycler with following parameters: 98 for 30 s, then five cycles of 98 for 10 s, 63 for 30 s, 72 for 30 s and final extension at 72 for 5 min. The products were size selected for SA1 +SA2+ SA cryptic+ unprocessed RNA (~ 391 to 800 bp) or exclusively for SA1 + novel 3'SS (~541 bp) and purified using gel extraction minelute kit (Qiagen). This was used as a template for second round of PCR with illumine compatible forward (F8) and barcoded reverse primer (R3) for another 5 cycles with cycling condition as mentioned above. To reduce size bias and stop PCR saturation, the appropriate number of cycle required was determined by qPCR. Following PCR with additional number of cycle as determined by qPCR, 10 % of products was run on 2% agarose gel to check for the expected size of amplicon. The remainder of reaction was purified in two ways: One library was prepared by isolating all transcripts (library A) using mini-elute PCR purification kit (Qiagen) and was used to calculate general statistics. A second library (library B) was prepared by selectively isolating sizes ~239 to 289 by gel and was used to determine odds ratio of SA1 use. Concentration and quality of each library was determined using qPCR and tape station.
Sequencing libraries for minigenes (plasmid) was performed as for RNA library as above by replacing the template cDNA with 100 ng of plasmid DNA library.

Minigene sequence (cloned into pCDNA3.1+)

The pDNA3.1-minigene library was prepared by serial cloning of two fragments (amplified from the 3'SS minigene library into at NheI-KpnI and EcoR1-Xba1 sites. Minigene variants (at -1, -2 and 0) were constructed from this construct by annealing of forward and reverse oligos and ligating into KpnI-EcoRI sites.

```
gctagcTCGCCACCATGGTGTCCAAGGGCGAGGAGCTGTTCACCGGGGTGGTGCCCATCCTGGTCGAGCTG
GACGGCGACGTAAACGGCCACAAGTTCAGCGTCAGCGGCGAGGGCGAGGGCGATGCCACCTACGGCAAACT
GACCCTGAAGTTCATCTGCACCACCGGCAAGCTGCCCGTGCCCTGGCCCACCCTCGTGACCACCTTCGGCT
ACGGCCTGATGTGCTTCGCCCGCTACCCCGACCACATGAAGCAGCACGACTTCTTCAAGTCCGCCATGCCC
GAAGGCTACGTCCAGGAGCGCACCATCTTCTTCAAGGACGACGGCAACTACAAGACCCGCGCCGAAGTGAA
GTTCGAGGGCGACACCCTCGTGAACCGCATCGAGCTAAAGGGCATCGACTTCAAGGAGGACGGCAACATCC
TGGGGCACAAGCTGGAGTACAACTACAACAGCCACAACGTCTATATCATGGCCGACAAGCAGAAGAACGGC
```

ATCAAAGTGAACTTCAAGATCCGCCACAACATCGAG**gt**aagttatcaccttcgtggctacagagtttcctt
atttgtctctgttgccggcttatatggacaagcatatcacagccatttatcggagcgcctccgtacacgct
attatcggacgcctcgcgagatcaatacgtataccagctgccctcgatacatgtcttggacggggtcggta
ccgatatcgtatagtatgcagtagtt**ctaac**atcggcgcttggatctgatctcaac**ag**ggtttcattccta
gccgtttgttttgggatgaattccacatatagacacgcgagcacccatcttttatagaatgggtagaaccc
gtcctaaggactcagattgagcatcgtttgcttctcgagtactacctggtac**ag**atgtctcttcaaac**ag**G
ACGGCAGCGTGCAGCTCGCCGACCACTACCAGCAGAACACCCCCATCGGCGACGGCCCCGTGCTGCTGCCC
GACAACCACTACCTGAGCTACCAGTCCGCCCTGAGCAAAGACCCCAACGAGAAGCGCGATCACATGGTCCT
GCTGGAGTTCGTGACCGCCGCCGGGATCACTCTCGGCATGGACGAGCTGTACAAGGACTGAtagtaaggcc
cattacctgcgcagaacacagcggttcgactgtgccttctagttgccagccatctgttgtttgcccctccc
ccgtgccttccttgaccctggaaggtgccactcccactgtcctttcctaataaaatgaggaaattgcatcg
cattgtctgagtaggtgtcattctattctggggggtggggtggggcaggacagcaagggggaggattggga
agacaatagcaggcatgctggggatgcggtgggctctatggcttctgaggcgtctaga

AGTATGCAGTAGTTCTAACATCGGC

Coding regions of the two exons are in upper case shaded light blue, intron and non-coding sequence are in lower case.
5'SS (gt) is in bold black, SA1 in bold green, SAcryptic in bold blue and SA2 in bold purple.
SA1 Branchpoint (BP) is in bold red (nucleophilic adenosine underlined).
Cloning sites are highlighted in light green: NheI(gctagc), KpnI(ggtacc), EcoRI(gaattc) and XbaI(tctaga)

Bioinformatics Analysis

1. Sequence preprocessing and Splice junction determination

Informatic analysis was generally modeled after previous report(1) with the following additional details/ changes. To align the transcript reads, a reference minigene library was first constructed from the 3'SS minigene plasmid library using Starcode algorithm (https://github.com/gui11aume/starcode).
The 20 bp unique sequence in the second exon was determined from the read 2 (R2) of paired Illumina reads and used a barcode to identify minigenes and to match them to transcripts.
Transcripts (from R1 of transcript library) were aligned to minigene sequences using the BLASTn algorithm (blast.ncbi.nlm.nih.gov) using the parameters [*–task blastn –outfmt 6*].
Those without alignments or >5 mismatches were discarded.
Splice junctions identified by the alignment were adjusted if needed for variations in length of 25A or 25B length for consistency (a small fraction of minigenes had 25A region with less than 25 bp, and similarly some minigenes were noted to have less than 25 bp in 25B). After such adjustment, frequencies (Splice_fraction) for individual splice positions (SA1, SA2, SAcryptic as well as other minor splice positions and those in 25A/25B) were computed.

The splice fraction of each splice sites was determined using the formula

$$\text{Splice\_fraction} = \frac{\text{No. of transcripts for a splice position in a minigene}}{\text{Total transcripts in that minigene}}$$

2. Calculation of odds ratio (OR)

To determine odds ratio (OR) of individual 6-mers, methodology as described previously was followed(1). Briefly, all possible combinations of a 6mers from A,T,G and C were generated (n=4096). Degenerate sequences (25A and 25B) were scanned for the presence of each of the kmers. The minigenes with the presence of the kmer (atleast once) were then checked for presence or absence of SA1 splice site and divided into a SA1+ and SA1- set. Probability of SA1 use for each kmer was then determined using the formula:

$$p(SA1+) = \frac{\text{\# of minigenes using SA1 splice sites with the } kmer}{\text{Total minigenes with the } kmers}$$

Once p(*SA1*+) was determined the odds were calculated using the formula

$$\text{Odds}(SA1+) = \frac{p(SA1+)}{1-p(SA1-)}$$

Similarly, odds of SA1 not being used for each kmer can be calculated

$$p(SA1-) = \frac{\text{\# of minigenes not using SA1 splice with the } kmer}{\text{Total minigenes with the } kmers}$$

The ratio was calculated using the equation

$$\text{Odds Ratio} = \frac{\text{Odds}(SA1+)}{\text{Odds}(SA1-)}$$

Odds and Odds ratio were calculated separately for SF3B1WT and SF3B1K700E datasets for comparison. Log2OR was used for comparing OR_WT and OR_K700E.

3. **Branch Point(BP) Prediction and free energy**

BP prediction was performed using LabRanchoR (https://github.com/jpaggi/labranchor) by analyzing 70 bp upstream of the 3'SS and using the BP with highest probability as the likely BP for that minigene. RNA free energy for U2-BP interaction was predicted using RNAhybrid (https://bibiserv2.cebitec.uni-bielefeld.de/rnahybrid).

4. **Data visualization and preparation of figures.**

Figures were prepared using ggplot2 (https://cran.r-project.org/web/packages/ggplot2/). Wilcoxon rank sum test was performed using the wilcox.test function in R. Sequence logos were created using the Weblogo package(https://weblogo.berkeley.edu/logo.cgih)

SURVEYOR Assay:

5 million mESC were transfected with 10ug pX-459-V2 sgRNA or a control GFP plasmid using Lipofectamine® (Thermo Fisher Scientific) reagent according to manufacturer's

protocol. 24 hours after transfection cells were selected with 2 ug/mL Puromycin for 48 hours. After Puromycin selection, cells were grown to 50-60% confluence in complete growth medium. Cells were then harvested and genomic DNA (gDNA) was harvested from sgRNA and GFP transfected cells using Qiagen Puregene Core Kit B (Cat# 158467) according to manufacturer's protocol. The genomic locus expected to be targeted by pX-459-V2 sgRNA was amplified from gDNA by PCR amplification using the following primers: Forward 5' CTGTTAACAAATTACCATTGCCT 3' and Reverse 5' TCGGTTATTATCTTACTAGCAAC 3'. The presence of mismatches within the amplified fragment resulting from non-homologous end-joining (NHEJ) following the introduction of double-stranded breaks by sgRNA-directed Cas-9 nuclease activity was assayed using the SURVEYOR ® mutation detection kit (IDT Cat# 706025) according to the manufacturer's protocol.

GUIDE-seq:

Off-target activity of sgRNA-directed Cas9 nuclease was assessed by GUIDE-seq as described previously(2). Briefly, 5 million mESCs were co-transfected with a double stranded oligonucleotide (dsODN) and 4 ug of pX-459-V2 sgRNA or pX-459 V2 empty vector. 24 hours after transfection cells were selected with 2 ug/mL Puromycin for 48 hours. After selection, cells were allowed to grow to 50-60% confluence and gDNA was harvested using Qiagen Puregene Core Kit B (Cat# 158467) according to manufacturer's protocol. GUIDE-seq libraries were prepared using primers specific for the dsODN sequence and libraries were sequenced using an Illumina MiSeq system. Sequencing resulted in 33 million total reads and 16.5 million reads per sample. Guide seq analysis package was used to analyze the Guide seq data. GUIDE-seq enables genome-wide profiling of off-target cleavage by CRISPR-Cas nucleases. Reads were demultiplexed and PCR duplicates were removed using the Unique molecular index(UMI) barcode information. Reads were mapped to mouse genome reference (NCBI Build 37 /mm9) using BWA-MEM algorithm with default parameters. A sliding genomic window of size 10 bp with reads mapped to both + and- strands or to same strand but amplified with both forward and reverse tag-specific primers were flagged as double-strand DNA breaks(DSBs).25 bp of reference sequence flanking both sides of breakpoints were then aligned to target sequence GCTCAAGCCCCTATGGA using Smith-Waterman local-alignment algorithm. Off-target cleavage site with more than 6 mismatches to target sequence are filtered out.

REFERENCES:

1. Rosenberg AB, Patwardhan RP, Shendure J, Seelig G. Learning the sequence determinants of alternative splicing from millions of random sequences. Cell. 2015 Oct 22;163(3):698–711.
2. Tsai SQ, Zheng Z, Nguyen NT, Liebers M, Topkar VV, Thapar V, et al. GUIDE-seq enables genome-wide profiling of off-target cleavage by CRISPR-Cas nucleases. Nat Biotechnol. 2015 Feb;33(2):187–97.

Supplementary Table 1: Oligonucleotide sequences

NGS Alt 3'SS RNA and DNA library primers		
**Name**	**Strand**	**Sequence (5' – 3')**
2506-DEG.F1	Forward	TACACGACGCTCTTCCGATCTNNNNNNGATCCGCCACAACATCGAG
2507-DEG.F2	Forward	TACACGACGCTCTTCCGATCTNNNNNNNGATCCGCCACAACATCGAG
2508-DEG.F3	Forward	TACACGACGCTCTTCCGATCTNNNNNNNNGATCCGCCACAACATCGAG
2509-DEG.F4	Forward	TACACGACGCTCTTCCGATCTNNNNNNNNNGATCCGCCACAACATCGAG
2510-DEG.F5	Forward	TACACGACGCTCTTCCGATCTNNNNNNNNNNGATCCGCCACAACATCGAG
2511-DEG.F6	Forward	TACACGACGCTCTTCCGATCTNNNNNNNNNNNGATCCGCCACAACATCGAG
2512-DEG.R1	Reverse	TCGAACCGCTGTGTTCTGC
2513-LIB.F7	Forward	AATGATACGGCGACCACCGAGATCTACACTCTTTCCCTACACGACGCTCTTCCGATCT
2514-LIB.R2	Reverse	CAAGCAGAAGACGGCATACGAGATNNNNNNGTGACTGGAGTCGAACCGCTGTGTTCTGC
2678- LIB.F8	Forward	AATGATACGGCGACCACCGAGATCTACACTTGGACGGGGTCGGTGTTGATATCGTAT

PCR primers for co-amplification of SA1 and SA2		
**Name**	**Strand**	**Sequence (5' – 3')**
2518-CIT.F1	Forward	CTATATCATGGCCGACAAGCAG
2519-CIT.R1	Reverse	GGGTGTTCTGCTGGTAGTGGT

PCR primers for qRT-PCR			
**Name**	**Strand**	**Sequence (5' – 3')**	**Gene**
2129-FLAGSF3B1	Forward	GCTCTACCCCTGTGCTGACA	FLAG-SF3B1
2130-FLAGSF3B1-R	Reverse	TCGATTGCGCTCGTCGATTT	
2832- Neo-F1	Forward	CTTGCTCCTGCCGAGAAAGT	NEOMYCIN
2833-Neo-R1	Reverse	ATGCGATGTTTCGCTTGGTG	
2838-SA1-R1	Reverse	GGCTAGGAATGAAACCCTCGA	SA1 isoform
2839-SA2-R1	Reverse	CTGCACGCTGCCGTCCTCGA	SA2 isoform
519-GAPDH-F	Forward	ATCCACGAAACTACCTTCAACTC	GAPDH
520-GAPDH-R	Reverse	GAGGAGCAATGATCTTGATCTTC	

Minigene cloning primers		
**Name**	**Strand**	**Sequence (5' – 3')**
Mini-F1		CAAGCTAGCTCGCCACCATGGTG
Mini-R1		AACGGTACCGACCCCGTCCAAGACATG
Mini-F2		AATGAATTCCACATATAGACACGCGAGC
Mini-R2		GAGTCTAGACGCCTCAGAAGCCATAGAGC
Mini-F3		GTACGATATCGTATAGTATGCAGTAGTTCTAACATCGGC
Mini-R3		AGATCAGATCCAAGCGCCGATGTTAGAACTACTGCATACTATACGATATC
Mini-F4		GCTTGGATCTGATCTCAACAGGGTTTCATTCCTAGCCGTTTGTTTTGGGAT
Mini-R4		AATTATCCCAAAACAAACGGCTAGGAATGAAACCCTGTTG
Mini-F5		GTACGATATCGTATAGTATGCAGTAGTTCTGACATCGGC
Mini-R5		AGATCAGATCCAAGCGCCGATGTCAGAACTACTGCATACTATACGATATC
Mini-F6		GTACGATATCGTATAGTATGCAGTAGTTCTCACATCGGC
Mini-R6		AGATCAGATCCAAGCGCCGATGTGAGAACTACTGCATACTATACGATATC
Mini-F7		GTACGATATCGTATAGTATGCAGTAGTTCTTACATCGGC
Mini-R7		AGATCAGATCCAAGCGCCGATGTAAGAACTACTGCATACTATACGATATC
Mini-F8		GTACGATATCGTATAGTATGCAGTAGTTCCAACATCGGC
Mini-R8		AGATCAGATCCAAGCGCCGATGTTGGAACTACTGCATACTATACGATATC
Mini-F9		GTACGATATCGTATAGTATGCAGTAGTTCTAGCATCGGC
Mini-R9		AGATCAGATCCAAGCGCCGATGCTAGAACTACTGCATACTATACGATATC

Oligos for mESC characterization		
**Name**	**Strand**	**Sequence**
Surveyor	Forward	TGAAAGACTTCTCACGCAAATTT
Surveyor	Reverse	TAGTATAGTAGTTGGCATATTCT
mESC_integration	Forward	GCCACTGTTGATTGATGAAGA
mESC_integration	Reverse	GGCTGAGCACACCTTTAATT

Full length mSF3B1	Forward	CCTCTGTTGATGTCCCCTACAC
Full length mSF3B1	Reverse	CTGCTGCGCCTAGATTACCC
Grtp1	Forward	GGACGCTCTTGTTGGAAGGA
Grtp1	Reverse	CAGGGTCCACAACACACCAT
Actr1b	Forward	CCACAAAAGGACGAGGCTCT
Actr1b	Reverse	AGACTTGTGGATGGCGAAGG
Ptk7	Forward	TTAGGAAGCCCCAAGACAGC
Ptk7	Reverse	GTCCCGTCATACACCTCCAC

Supplementary Table 2: Western Blot Antibodies and Conditions

Primary Ab	Company	Catalogue #	Dilution	Time probed	T	Secondary Ab	Company	Catalogue #	Dilution	Time probed	T
Flag	SIGMA	F3165	1:1000	O/N	4°C	S anti M	GE	NXA931	1 :10000	1 hr	RT
Human SF3B1	ABCAM	ab170854	1:1000	O/N	4°C	G anti R	Rockland	611-1302	1 :10000	1 hr	RT

Abbreviations:

T Temperature
Ab Antibody
G Goat
R Rabbit
M Mouse
S Sheep
RT Room Temperature
O/N Overnight
